# ^90^Y Radioembolization: Telemedicine During COVID-19 Outbreak, Opportunity for Prime Time

**DOI:** 10.2967/jnumed.120.246389

**Published:** 2020-06

**Authors:** Lawrence Quek, Anbalagan Kannivelu, Uei Pua

**Affiliations:** * Tan Tock Seng Hospital 11 Jalan Tan Tock Seng, Singapore 308433 E-mail: lawrence_quek@ttsh.com.sg

**TO THE EDITOR:** Telemedicine is broadly defined as the use of audiovisual technology in the provision of medical care remotely (1). Recent developments in the COVID-19 outbreak necessitated the change of many conventional practices, to avoid cross-infection between health-care institutions and patients (2). To this end, in Singapore, the movement of doctors between institutions and cross-institutional referral of patients is discouraged, save for absolute essential life-saving services. As such, institutions that are dependent on external (non–in-house) medical expertise will be affected, risking nondelivery or suboptimal care to chronic care patients or complex treatment, such as in oncology. To this end, telemedicine can be expounded to bridge the gap in this time of contingency.

Our institution has used telemedicine to ensure continuity of care during this crisis in patients requiring ^90^Y selective internal radiation therapy (^90^Y SIRT). ^90^Y SIRT requires multidisciplinary involvement, including interventional radiologists (IR) for arteriography and nuclear medicine (NM) physicians for both dosimetry and procedural aspects of the therapy, which includes verification of the radioactive doses to be administered, set-up of the delivery system, and administration of the dose conjointly with the IR.

In our institution, our NM expertise from an outside institution was prohibited from being physically onsite due to the new restriction. To overcome this, before any planning of hepatic arteriography and intraarterial ^99m^Tc-macroaggregate albumin administration (^99m^Tc-MAA), the case history and recent cross-sectional images are electronically transmitted using a secured encrypted channel to the off-site NM physician, which will precipitate case discussion, either via secured text messaging or over teleconversation, for suitability of ^90^Y SIRT as well as the mapping considerations. Hepatic angiographic and scintigraphic images obtained after ^99m^Tc-MAA administration would be electronically shared for deciding the feasibility and arterial approach, calculation of the perfused territorial volumes, and finalizing the ^90^Y SIRT doses. Similarly, Bremsstrahlung scan images obtained after ^90^Y implantation would be electronically transmitted for confirming the localization of ^90^Y microspheres as per the treatment plan. In the procedural room, our unit uses videoconferencing (one with transport encryption) to ensure continuity of the service—the NM physician is “virtually” present to verify the configuration of the delivery system as well as appropriate sites of delivery of the ^90^Y and monitor radioactive therapy as the IR administers the dose—compliant with our national regulatory authority for radiation protection.

This retrospective study fulfills our institutional review board’s requirement for waiver to obtain informed consent. To date, we have treated 3 cases (all men; mean age, 66 y; range, 57–81 y) successfully without complications. These include 2 cases of hepatocellular carcinoma (HCC) and a liver dominant colorectal metastatic disease. Radioembolization was performed per standard methodology, delivering a radiation dose of 80–150 Gy to the hepatic parenchyma using glass microspheres (3); both the cases with HCC were treated with an additional boosted dose to the segment with the largest tumor (range, 6.3–9.0 cm) (4). Mean total radiation activity infused was 3.10 GBq ± 1.13 (median, 2.45 GBq; range, 2.4–4.4 GBq). Mean target perfused tissue dose was 129.1 Gy ± 49.1 (median, 129.5 Gy; range, 72–181.3 Gy). The mean number of vials used per treatment was 2. All patients were admitted to the hospital and discharged 1 d after the procedure.

Overall, use of telemedicine has allowed our institution to continue collaborating with our off-site NM physician partners in delivery of oncologic care to our patients during this outbreak, which currently shows no sign of any abatement in the near future. Recognizing the importance of telemedicine, the Society of Nuclear Medicine and Molecular Imaging (SNMMI) and the European Association of Nuclear Medicine (EANM) provided guidelines for tele-nuclear medicine in interpretation of routine NM studies at a remote location, interpretation of emergency studies in an on-call setting, and consultation (5). This outbreak may spark a wider adoption of tele-nuclear medicine in the post–COVID-19 era—not just in diagnosis and therapy but also in education for developing nations with limited access to formal training in NM.

## DISCLOSURE

No potential conflict of interest relevant to this article was reported.
